# The Joint Vasculitis Registry in German-speaking countries (GeVas) – a prospective, multicenter registry for the follow-up of long-term outcomes in vasculitis

**DOI:** 10.1186/s41927-021-00206-2

**Published:** 2021-07-31

**Authors:** Christof Iking-Konert, Pia Wallmeier, Sabrina Arnold, Sabine Adler, Kirsten de Groot, Bernhard Hellmich, Bimba F. Hoyer, Konstanze Holl-Ulrich, Gabriele Ihorst, Margit Kaufmann, Ina Kötter, Ulf Müller-Ladner, T. Magnus, Jürgen Rech, Fabian Schubach, Hendrik Schulze-Koops, Nils Venhoff, Thorsten Wiech, Peter Villiger, Peter Lamprecht

**Affiliations:** 1 Sektion Rheumatologie, III Med. Klinik, Universitätsklinikum Hamburg Eppendorf, Hamburg, Germany; 2 Department of Rheumatology and Clinical Immunology, University of Lübeck, Lübeck, Germany; 3 SimZentrum Erfurt, Erfurt, Germany; 4 Med Klinik III, Innere Medizin, Rheumatologie, Nephrologie, Sana Klinikum Offenbach/Main, Offenbach, Germany; 5 Klinik für Innere Medizin, Rheumatologie und Immunologie medius KLINIK Kirchheim, Kirchheim, Germany; 6 Rheumatologie/klinische Immunologie und Exzellenzzentrum Entzündungsmedizin, Klinik für Innere Medizin I, UKSH Campus Kiel, Kiel, Germany; 7 Pathologie Hamburg, Labor Lademannbogen MVZ GmbH, Hamburg, Germany; 8 Clinical Trials Unit, Faculty of Medicine, Medical Center – University of Freiburg, University of Freiburg, Freiburg, Germany; 9 Sektion Rheumatologie, III Med. Klinik, Universitätsklinikum Hamburg Eppendorf und Klinikum Bad Bramstedt, Bad Bramstedt, Germany; 10 Abt. Rheumatologie und Klinische Immunologie, JLU Giessen, Campus Kerckhoff, Giessen, Germany; 11 Neurologische Klinik, Universitätsklinikum Hamburg Eppendorf, Hamburg, Germany; 12 Uni-Klinikum Erlangen, Department of Internal Medicine 3 - Rheumatology and Immunology, Friedrich-Alexander University (FAU) Erlangen-Nürnberg and Universitätsklinikum Erlangen, Erlangen, Germany; 13 Division of Rheumatology and Clinical Immunology, Department of Internal Medicine IV, Ludwig-Maximilians-University Munich, Munich, Germany; 14 Department of Rheumatology and Clinical Immunology, Faculty of Medicine, Medical Center – University of Freiburg, University of Freiburg, Freiburg, Germany; 15 Sektion Nephropathologie, Institut für Pathologie, Universitätsklinikum Hamburg-Eppendorf, Hamburg, Germany; 16 University Hospital and University of Bern, Bern, Switzerland

**Keywords:** Vasculitis, GeVas, ANCA, Prospective, Registry, Giant cell arteritis, Long term, Outcome, Therapy

## Abstract

**Background:**

Vasculitides comprise a group of rare diseases which affect less than 5 in 10.000 individuals. Most types of vasculitis can become organ- and life-threatening and are characterized by chronicity, high morbidity and relapses, altogether resulting in significant morbidity and mortality. Previous studies have been either monocentric or mainly retrospective – studies with a prospective design mostly consisted of rather small cohorts of 100 to 200 patients.

The aim of the Joint Vasculitis Registry in German-speaking countries (GeVas) is to record all patients who have been recently diagnosed with vasculitis or who have changed their treatment due to a relapse (inception cohort). In GeVas, data are collected prospectively in a multicenter design in Germany, Austria and Switzerland. By this approach, courses of vasculitis and their outcomes can be monitored over an extended period.

**Methods:**

GeVas is a prospective, web-based, multicenter, clinician-driven registry for the documentation of organ manifestations, damage, long-term progress and other outcomes of various types of vasculitis. The registry started recruiting in June 2019. As of October 2020, 14 centers have been initiated and started recruiting patients in Germany. Involvement of sites in Austria and the German-speaking counties of Switzerland is scheduled in the near future.

**Discussion:**

In June 2019, we successfully established a prospective multicenter vasculitis registry being the first of its kind in German-speaking countries. The participating centers are currently recruiting, and systematic analysis of long-term vasculitis outcomes is expected in the ensuing period.

**Trial registration:**

German Clinical Trials Register (Deutsches Register Klinischer Studien): DRKS00011866. Registered 10 May 2019.

## Background

Vasculitides are rare diseases affecting less than 5 in 10.000 people. In general, the vast majority of vasculitides cannot be cured. Thus, they are characterized by chronicity, high morbidity, mortality, and a high tendency for relapse. Most of them are potentially organ- and life-threatening.

In contrast to clinical trials that include selected patient subtypes and often have a short follow up, cohort or registry studies provide important insight on disease presentation and long-term outcomes in a broad and representative population of patients. The significance of most of the earlier cohort studies in vasculitis is limited by various methodological shortcomings such as monocentric patient recruitment, retrospective design and limited number of outcome parameters [1–6]. Furthermore, small cohort studies of no more than 100 to 200 patients have been conducted following response to immunosuppressive therapies with limited follow-up over time. Results of these studies were subsequently supported by follow-up studies with a narrow thematic focus [7–13]. The incidence and prevalence of various types of vasculitis in local regions were calculated based on data of particular university and reference centers [14–16]. In contrast to registry-based data, these studies do not support conclusions regarding initial and cumulative organ involvement, organ damage, long-term progress, and outcomes in various types of vasculitis [17].

Therefore, there is a persistent need for registries documenting disease manifestations and courses, treatment, comorbidities, and long-term outcomes of vasculitis patients in a standardized, multicenter, and prospective manner. Through establishing a registry in German-speaking countries, these rare diseases will be systematically and prospectively recorded for the first time in this European region, thereby enabling the standardized documentation of disease outcomes under the supervision of physicians specialized in vasculitis patient care over an extended period. Furthermore, it is possible to perform comparative analyses on a much larger scale than is possible in monocentric studies.

These data will specifically shed light on how guidelines and standards of treatment are implemented in local centers dealing with the treatment of vasculitis patients, and thus provide insight into the current state of medical care. Analyzing characteristics of the diseases and their outcomes will also be relevant in devising hypotheses and treatment objectives for new treatments for investigator-initiated clinical trials (IITs). Data from the registry may also serve as a basis for identifying needs for improving the structure of healthcare with regard to the specific needs of vasculitis patients in German-speaking countries. Lastly, GeVas will facilitate comparative analyses with other European vasculitis registries.

Here, we report the protocol, methodology, and status of this registry.

## Methods

### Objectives

To record all patients who have been recently diagnosed with systemic or single-organ vasculitides according to the Chapel Hill Consensus Conference (CHCC) nomenclature and definitions, or who have changed their treatment due to a relapse, in a prospective, web-based, clinician-driven, transregional, multicenter registry, and to document long-term disease outcomes in a standardized and systematic manner.

### Trial design

Prospective, web-based, multicenter disease registry.

### Study setting

This registry study is conducted at in- and out-patient care centers involved in the management of vasculitis patients (licensed medical specialists, specialized out-patient departments and wards of secondary and tertiary care facilities) in Germany, Austria and Switzerland. It is intended to include as many centers specialized in vasculitis care and treatment as possible in order to achieve a nationwide coverage of the target patient population. Such centers take care of patients with vasculitis on a regular basis and participate in vasculitis research and/or clinical trials. After obtaining appropriate ethics approvals, the study started by successively initiating centers in Germany. Centers in Austria and German-speaking parts of Switzerland will follow and join the registry.

### Eligibility criteria

Patients with any form of systemic or single-organ vasculitis as defined by the CHCC nomenclature and definitions are included [18]. Patients are registered if they have been newly diagnosed with the disease no more than 6 months before the first visit or if they have had a relapse with change of immunosuppression. Due to the lack of current diagnostic criteria for classifying vasculitis, the following criteria are used for inclusion of patients into the registry [18, 19]: Classification criteria employed in national and international studies, i.e. the American College of Rheumatology Classification Criteria, the nomenclature and definitions of the Chapel Hill Consensus Conference, and the MIRRA criteria for eosinophilic granulomatosis with polyangiitis (EGPA) [18–20]. Moreover, anti-neutrophil cytoplasmic autoantibody-associated vasculitis (AAV) and polyarteritis nodosa can be classified into the respective category using clinical surrogate endpoints following the European Medicines Agency (EMA) algorithm I case histological confirmation of the diagnosis is not possible [21].

Table 1 shows the vasculitis entities eligible to be included in this registry.

**Table 1:** Vasculitis entities eligible for inclusion in the GeVas registry

Vasculitis entities	
**1. Large vessel vasculitis**	
• Giant Cell Arteritis • Takayasu Arteritis	
**2. Medium size vessel vasculitis**	
• Polyarteritis nodosa • Kawasaki disease	
**3. Small vessel vasculitis**	
**ANCA-associated vasculitides**	
• Microscopic Polyangiitis • Granulomatosis with Polyangiitis • Eosinophilic Granulomatosis with Polyangiitis	
**Immune complex vasculitis**	
• Anti-GBM(glomerular basement membrane) disease • Cryoglobulinemic vasculitis • IgA vasculitis • Hypocomplementemic urticarial vasculitis (Anti-C1q vasculitis)	
**4. Variable Vessel Vasculitis**	
• Behcet’s disease • Cogan’s Syndrome	
**5. Single Organ Vasculitis**	
• Cutaneous (leukocytoclastic) small vessel vasculitis • Cutaneous arteritis • Primary central nervous system vasculitis • Isolated aortitis • Others	
**6. Vasculitis associated with systemic disease**	
• Lupus vasculitis • Rheumatoid vasculitis • Sarcoid vasculitis • Others	
**7. Vasculitis associated with probable etiology**	
• Hepatitis C virus-associated cryoglobulinemic vasculitis • Hepatitis B virus-associated polyarteritis nodosa • Syphilis-associated vasculitis • Drug-associated immune complex vasculitis • Drug-associated ANCA-associated vasculitis • Cancer-associated vasculitis • Others	

### Interventions

Not applicable. This registry is purely observational in nature, and relies on the documentation of routine data only. Hence, no study-specific interventions are performed.

### Outcomes

The outcomes assessed in this registry are listed in Table 2. An explicit distinction between primary and secondary outcomes is not made, but a list of equivalent outcomes has been defined.

**Table 2:** List of outcomes for the GeVas registry

Outcomes	
Demography / Consent	
Initial manifestation / Relapse as defined by EULAR guidelines / Change of treatment	
Comorbidity as outlined in the eCRF during initial manifestation and over the course of disease progression as defined by EULAR guidelines (e.g. diabetes, hypertension, atherosclerotic vasculopathy, osteoporosis)	
Vasculitis entity and formal diagnosis	
Disease activity (as defined in 25)	
Clinical manifestations related to active vasculitis features related to active vasculitis features (e.g. general symptoms, eyes, ENT, lung/chest, renal symptoms, PNS/CNS features etc.)	
Laboratory tests e.g. CRP, ANCA etc.	
Dialysis yes / no // renal transplantation yes / no	
Treatment and response to immunosuppressive therapy (as defined in 25)	
Frequency and types of infectious complications such as pneumonia, urinary tract infections	
Occurrence of comorbidity during disease progression	
Cancer yes/no	
Pregnancy yes/no	
Death incl. Date and cause of death	
BVAS Score / BVAS items (BVAS: Birmingham Vasculitis Activity Score)	
VDI Score / VDI items (VDI: Vasculitis Damage Score)	

### Participant timeline

The visit schedule includes a baseline visit at initial registration (visit 1), at which the diagnosis and the introduction of remission-inducing treatment is documented. A second visit is scheduled after 3 ± 2 months, and a third after 6 ± 3 months, in order to document the switch from remission-inducing to remission-maintaining treatment usually achieved after 3–6 months. Afterwards, visits are scheduled at intervals of 6 months in order to monitor treatment regularly. This visit schedule meets the standard procedures for managing vasculitis patients and is in accordance with the proposed European Vasculitis Society (EUVAS) recommendations for national registries currently undergoing further specification [22–25]. In case of new relapse, complications or other incidents occurring between two visits, these can be entered as unscheduled visit in between or at the next follow-up visit. Registered patients will be followed up for at least 36 months after initial diagnosis; however, this period can be extended.

### Sample size

The number of patients to be included into this registry is not based on a formal sample size calculation, as the purpose of this registry is to obtain an exhaustive documentation of patients with a recent onset or relapse of vasculitis. Given an incidence of 100–200 per 10^6^ inhabitants per year and a prevalence of 400–1.000 per 10^6^ inhabitants for the group of vasculitides [3, 4, 26, 27], it is estimated that approximately 1–5 patients per month will be enrolled into the registry by each center. The period for registering patients, as well as the number of patients, is not limited to ensure a description of the long-term course of the disease that is as accurate and comprehensive as possible.

### Recruitment

All patients with a newly diagnosed vasculitis, or with a treatment change due to relapse, will be screened for inclusion into the registry at the respective study site. If a patient is eligible, she or he will be informed about the study and asked for written informed consent. As vasculitis patients are usually cared for by specialized consulting physicians or specialized out-patient departments or hospitals, we aim to include as many of these specialized centers as possible into the registry.

Since its initiation in June 2019, our registry has included 14 sites in Germany and documented more than 100 vasculitis patients. GeVas is driven by an active and quickly growing research community. Further sites across Germany, Austria and Switzerland are being recruited. As of November 2020, more than 20 additional sites in the three countries have expressed their interest to participate.

### Data collection methods and data management

Data collection takes place via a web-based, electronic case report form (eCRF) in RDE-LIGHT (RDE = Remote Data Entry), a system based on HTML and Javascript that has been developed in-house by the Clinical Trials Unit (CTU) of the Medical Center, University of Freiburg. The data center of the Medical Center, University of Freiburg, runs the system (software, MySQL-database and web server), which is subject to strict access controls. Data transfer is encrypted (https, SSL certificate). RDE-LIGHT is validated according to GAMP5 and is GCP-compliant, e.g. presence of an audit trail. Furthermore, all data is backed up on a daily basis and archived regularly. User account management is controlled centrally by the CTU in Freiburg. Appropriate technical and organizational measures are implemented to comply with EU General Data Protection Regulation (GDPR) and other applicable data protection legislation.

The participating centers receive a video tutorial and a manual in print for accessing and using the eCRF. The participating centers enter the appropriate routine data from the medical records of the patients into the eCRF via a standard web browser. The eCRF is organized into specific sections (demographics, vasculitis entity, clinical features, immunosuppressive treatment, etc.) which correspond to the list of outcomes and in which the respective parameters are recorded in a standardized form. Participating centers can view their own data and export them in DSV format (delimiter-separated values).

Data recording is in pseudonymous form only, that is, each patient is assigned a patient identification code in the registry before any data entry takes place. The patient ID code is assigned by the respective participating site and can be decoded by this site only.

### Statistical methods

The primary objective of the study is to register all patients who have been diagnosed recently with vasculitis or who have changed their treatment due to relapse, and to document their long-term disease course. The statistical analysis is therefore solely descriptive.

Initially, the quality of data will be described, i.e. the number of documented patients and the completeness of the data. Demographic information, incidences of different types of vasculitis, organ damage, comorbidity and additional disease-specific symptoms upon initial diagnosis will be analyzed in order to describe the patient cohort. Time-to-event endpoints, such as overall survival time, time to treatment response and time to relapse, are estimated and described using Kaplan-Meier methodology.

Beside the median, the corresponding 95% confidence intervals for the respective time to event will be presented. Continuous variables will be presented with number of observations, mean, standard deviation, 25% quantile, median, 75% quantile as well as minimum and maximum. Categorical variables will be presented by absolute and relative frequencies.

Analyses will be performed for all patients. Depending on the research question and the number of available observations, subgroup analyses by type of vasculitis and center may be performed in addition.

The first analysis will be conducted 12 months after the registration of the first patient and yearly analyses are envisaged. Analyses relating to specific research questions can be conducted as required.

### Study management and oversight

PL as the coordinating investigator, and CIK as his deputy, take the scientific project lead and oversee the design and conduct of the study. Furthermore, the conduct and management of the registry is supported by a steering committee, several working groups and a CTU. The steering committee assists and advises the coordinating investigators in major and overarching scientific issues, agrees on the final protocol and amendments, and reviews the study progress. Working groups have been established for the following topics: ANCA-associated vasculitis, giant cell arteritis, CNS vasculitis, Behcet’s disease, IgA vasculitis, nephrology, and pathology. The working groups support the coordinating investigators and the steering committee by complementing the registry design and developing scientific questions for their respective focus. Finally, the CTU is responsible for the administrative and regulatory project management, e.g. preparation of submissions to independent ethics committees, data management, e.g. design, validation and maintenance of case report forms, hosting of the study database, user support, and planning and performance of statistical analyses.

### Data monitoring

Currently, measures for monitoring data quality after entry, such as a systematic query management or clinical monitoring are currently not implemented. It is intended to implement such measures in case of additional funding being obtained. The establishment of a data safety monitoring board (DSMB) is not required due to lack of study-specific interventions.

### Interim analyses and stopping guidelines

As specified above, analyses of registry data are scheduled on an annual basis. There are no study-specific stopping guidelines other than withdrawal of consent of individual participants.

### Harms

Study-specific harms are not to be expected as no study-specific interventions are carried out and only routine data are documented. However, suspected side effects or complications of immunosuppressive therapy will be reported on the appropriate eCRF form. Periodically, listings will be prepared and missing entries will be inquired.

#### Auditing

Audits may be performed according to local quality assurance requirements of the participating centers at any time.

#### Ethics and dissemination

##### Research ethics approval

The study was approved by the Ethics committee (EC) of the University of Lübeck (EC reference number: 16–306) as well as by the responsible EC of each participating center.

### Protocol amendments

Substantial amendments to the registry protocol (e.g. changes to inclusion criteria, outcomes, analyses) will only be implemented after ethics approval has been obtained.

### Consent or assent

The patients must give their written informed consent for data to be entered into the registry. If the patient is incapable of giving consent, a legal representative must give his or her written informed consent based on the patient’s presumed will.

### Access to data

The coordinating investigators and the CTU hosting the database will have access to the full dataset. Each registry site has access to its own data as outlined above (see: Data collection methods and data management). Authors of future publications of registry analyses will be granted access to data upon request to the steering committee.

### Ancillary and post-trial care

As no harms are to be expected from the participation in this registry, no specific arrangements are required for study-specific ancillary care or post-study treatment. During as well as after registry participation, decisions concerning the medical care of patients will be based on clinical considerations only.

### Dissemination policy

The results of the pre-scheduled annual analyses of this registry will be submitted for publication in scientific journals and presented at academic conferences regardless of outcome. No contractual restrictions exist regarding publication of study results. Authors of the main publications shall be all those who fulfil the ICMJE recommendations on authorship.

## Discussion

In June 2019, the GeVas registry started recruiting vasculitis patients as the first prospective and multi-center registry in German-speaking countries. Since then, additional study centers have successively been initiated. As of 31st October 2020, 169 patients have been included in 14 participating centers. More centers will be initiated in Germany, in Austria and in the German-speaking parts of Switzerland in the near future.

By this multicenter registry, disease manifestations, methods and procedures of treatment, comorbidities and long-term outcomes of vasculitis patients can be documented in a prospective manner. As discussed above (see: background), earlier studies were limited by monocentric patient recruitment, retrospective design and methodology, and by a limited number of outcomes measured as well as by a limited numbers of recruited patients. Moreover, Pagnoux et al. showed, that patients with AAV in RCTs and those in observational cohorts show important differences [28]; therefore there is a need for more high quality real life data.

GeVas will record vasculitis patients systematically and prospectively, thus – in the long run – it will provide important information for improving the care and outcomes of vasculitis patients.

This is an ongoing project; a first status report has already been presented at the congress of the German Society for Rheumatology (DGRh) in September 2020. Subsequent analyses of registry data are scheduled on an annual basis as outlined above (See: Statistical Methods).

## Data Availability

Data sharing is not applicable to this article as no datasets were generated or analyzed during the current study.

## Abbreviations

CHCC: Chapel Hill Consensus Conference
CTU: Clinical trials unit
EC: Ethics committee
eCRF: Electronic case report form
